# Learning Curve of Pentaspline Pulsed‐Field Ablation With Posterior Wall Isolation for Persistent Atrial Fibrillation

**DOI:** 10.1111/jce.70515

**Published:** 2026-09-27

**Authors:** Atsuhiko Yagishita, Kazuma Iimura, Aika Iijima, Mari Amino, Yuji Ikari, Koichiro Yoshioka

**Affiliations:** ^1^ Department of Cardiology Tokai University School of Medicine Isehara Kanagawa Japan

**Keywords:** atrial fibrillation, learning curve, posterior wall isolation, pulmonary vein isolation, pulsed‐field ablation

## Abstract

**Background:**

Pentaspline pulsed‐field ablation (PFA) has emerged as a novel modality for pulmonary vein isolation (PVI) and posterior wall isolation (PWI) in persistent atrial fibrillation (AF). However, the learning curve associated with combined PVI + PWI and the contribution of anatomical factors to acute lesion completeness remain incompletely defined.

**Methods:**

We analyzed 182 consecutive patients with persistent AF undergoing fluoroscopy‐ and intracardiac echocardiography–guided PFA. Acute incomplete lesions were defined as residual conduction requiring additional PFA applications after systematic remapping. Learning phases were identified using segmented logistic regression and sequential block analysis. Multivariable models evaluated procedural phase, left atrial volume (LAV), and posterior wall geometry.

**Results:**

A learning curve was observed, with the greatest reduction in incomplete lesion formation occurring during the first 20 procedures. Incomplete lesions occurred in 55% of cases 1–20 (early phase), 32% of cases 21–160 (intermediate phase), and 4.8% of cases ≥ 161 (late phase). Procedural phase was the dominant determinant of incomplete lesions, with approximately 24‐fold and 9‐fold higher odds during the early and intermediate phases, respectively. Larger LAV showed a modest independent association (OR 1.009 per mL), whereas posterior wall geometry was not independently associated. Mid‐term arrhythmia recurrence did not differ across phases, although follow‐up duration was shorter in the late phase.

**Conclusions:**

Lesion completeness during pentaspline PFA with PVI + PWI improves progressively with increasing procedural experience. Although larger LAV may contribute modestly to incomplete lesion formation, procedural experience appears to play the predominant role. Systematic electroanatomical remapping remains important for identifying and correcting incomplete lesions even after substantial procedural experience has been accumulated.

## Graphical abstract

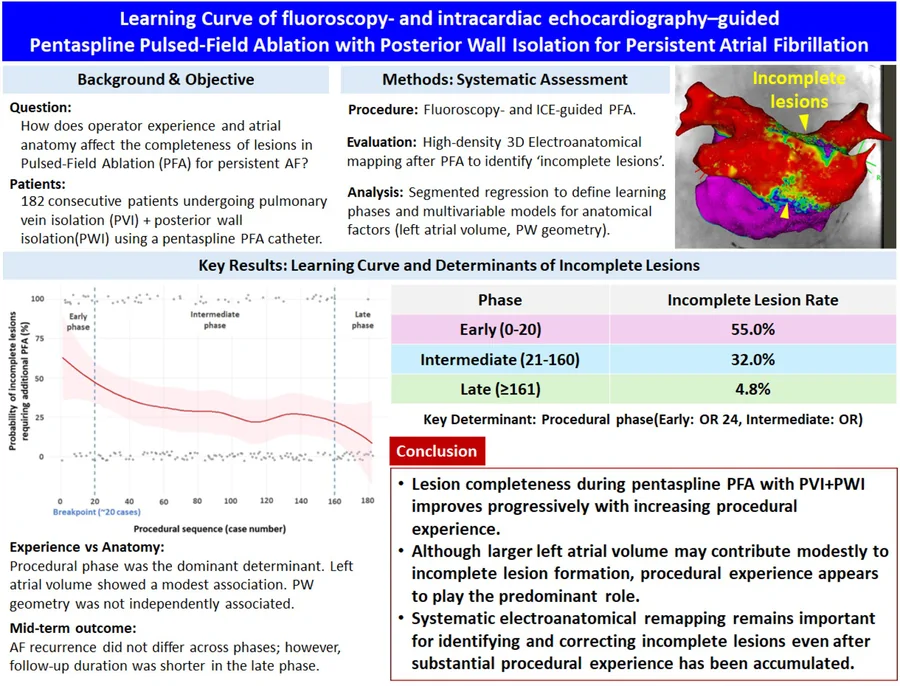

**Abbreviations**
3D: three‐dimensional
AF: atrial fibrillation
AT: atrial tachycardia
ICE: intracardiac echocardiography
LA: left atrium
LAV: left atrial volume
PFA: pulsed field ablation
PV: pulmonary vein
PVI: pulmonary vein isolation
PWI: posterior wall isolation

## 1 Introduction

Atrial fibrillation (AF) is the most common sustained arrhythmia and is associated with an increased risk of cardiovascular events and stroke, highlighting the importance of early and effective treatment strategies [1]. Catheter ablation has become an established therapy for AF, with pulmonary vein isolation (PVI) serving as the cornerstone of rhythm control strategies [2]. However, achieving durable lesion sets—particularly in patients with persistent AF—remains challenging. In this population, additional substrate modification such as posterior wall isolation (PWI) is frequently performed to improve outcome, although incomplete lesion formation and residual conduction across the posterior wall remain common and may contribute to arrhythmia recurrence [3, 4].

The introduction of pulsed‐field ablation (PFA), a nonthermal ablation modality based on irreversible electroporation, has reshaped contemporary AF ablation by offering myocardial selectivity and a favorable safety profile [5, 6]. Early clinical trials have demonstrated the safety and feasibility of pentaspline PFA systems for PVI and PWI [7, 8, 9, 10]. Nevertheless, lesion formation in PFA depends on electric field distribution, catheter geometry, and tissue contact, which may introduce variability in lesion continuity during the early adoption phase [11]. Indeed, recent observations using high‐density three‐dimensional (3D) electroanatomical mapping have demonstrated that incomplete PWI may occur despite apparently complete lesion sets under fluoroscopic and intracardiac echocardiography (ICE) guidance during early PFA experience [12]. These findings highlight the importance of systematically assessing lesion continuity and raise the possibility that procedural experience may play a key role in achieving consistent lesion completeness.

Despite the rapid global adoption of PFA, the procedural learning trajectory required to achieve stable lesion formation has not been quantitatively characterized. In particular, the learning curve of fluoroscopy‐ and ICE–guided pentaspline PFA for combined PVI and PWI in patients with persistent AF remains poorly defined. Furthermore, the relative contribution of anatomical factors—such as left atrial size or posterior wall morphology—to acute lesion continuity in the context of high‐density 3D electroanatomical remapping has not been systematically investigated. Therefore, in the present study, we sought to characterize the procedural learning curve of pentaspline PFA for PVI and PWI in patients with persistent AF and to identify anatomical determinants of acute incomplete lesions assessed by 3D electroanatomical mapping.

## 2 Materials and Methods

### 2.1 Study Population

In this retrospective cohort study using prospectively collected data, we consecutively enrolled 182 patients with persistent AF who underwent fluoroscopy‐ and ICE‐guided pentaspline PVI with PWI at our institution between December 2024 and November 2025. Patients were excluded if they underwent PVI alone without PWI, had a history of prior catheter ablation, had insufficient post‐procedural mapping data for lesion assessment, or lacked adequate follow‐up data for outcome evaluation. The study was conducted in accordance with institutional ethical standards. All patients provided written informed consent before undergoing the procedure. The study adhered to the ethical principles of the Declaration of Helsinki and was approved by the Institutional Ethics Committee of Tokai University Hospital (approval no. 25R209).

### 2.2 Catheter Ablation Procedure

The absence of atrial thrombus was confirmed using transesophageal echocardiography or computed tomography. The procedures were performed under conscious sedation with uninterrupted oral anticoagulation. Right femoral venous access was obtained, and a steerable dual‐decapolar electrode catheter (6 F BeeAT; Japan Lifeline Co. Ltd. Tokyo, Japan) was introduced into the coronary sinus, allowing simultaneous recording of the right atrium. A 100 IU/kg heparin bolus was administered after inserting the vascular sheaths. Bipolar electrogram recordings were filtered between 30 and 500 Hz and displayed on a commercially available electrophysiological recording system (Nihon Kohden Corporation, Tokyo, Japan). Heparinized saline was infused to maintain an activated clotting time of 350–400 s. Transseptal puncture was performed under ICE guidance using the SoundStar catheter (Biosense Webster, Irvine, CA, USA), with either a radiofrequency needle (NRG Transseptal Needle; Baylis Medical, Montreal, QC, Canada) or the VersaCross transseptal system (Baylis Medical, Montreal, QC, Canada), followed by advancement of a dedicated steerable sheath (FARADRIVE, Boston Scientific, Marlborough, MA, USA) into the left atrium. In patients without a history of glaucoma or benign prostatic hyperplasia, intravenous atropine sulfate (1 mg) was administered prophylactically prior to PFA to reduce vagal reactions. All ablation procedures were performed under fluoroscopic guidance using the penta‐spline PFA catheter system (FARAWAVE, Boston Scientific). In patients with paroxysmal AF, PVI was performed alone. In those with persistent AF, PVI combined with PWI of the left atrium was conducted. The PFA procedures followed a standardized protocol provided by the manufacturer. The FARAWAVE catheter was deployed in either a “basket” or “flower” configuration to achieve circumferential contact with the PV ostia. Pulsed field energy was delivered using the FARASTAR generator, typically at a setting of 2 kV with a pulse duration of 2 ms, delivered in bipolar mode. During PVI, two applications were delivered in one orientation of the basket or flower configuration, followed by catheter rotation to ensure that all splines covered a different segment. This sequence was repeated, for a total of eight applications per vein, in accordance with the manufacturer's recommendations. For PWI, the catheter was advanced using a continuous sequential deployment strategy with overlapping applications along the roof and inferior posterior wall, forming a contiguous lesion set during a single uninterrupted workflow (Supporting Information S1: Figure 1). After the application at the left superior pulmonary vein (LSPV), the catheter was advanced along the roof toward the right superior pulmonary vein (RSPV), followed by applications at the RSPV and right inferior pulmonary vein (RIPV), and subsequently extended toward the left inferior pulmonary vein (LIPV) with overlapping deployments to create a contiguous posterior wall lesion set. At each application site, two 2‐kV pulses were delivered, with 50% overlap between adjacent application sites to ensure lesion continuity. ICE was used throughout the procedure to confirm catheter‐tissue contact and stability, thereby optimizing energy delivery and lesion formation. No esophageal temperature monitoring was used, as PFA is considered nonthermal in nature. Although three operators were involved, one of the operators served as the senior operator and directly supervised all procedures, guiding catheter positioning and energy delivery during all applications to ensure procedural consistency. This approach ensured procedural consistency and allowed the learning curve to be evaluated at the procedural level rather than at the individual operator level. Post‐ablation, a detailed electroanatomic shell and voltage map of the left atrium were obtained during high right atrial pacing, with a pacing cycle length of 600 ms using a multielectrode mapping catheter (OctaRay Nav; Biosense Webster) and the CARTO3 version 7 ConfiDENSE Module (Biosense Webster) to assess incomplete lesion formation. Bipolar voltage maps were constructed using a voltage threshold of 0.05–0.5 mV. Point acquisition was performed using the nominal ConfiDENSE settings, including the default fill threshold (interpolation) and point acquisition filters for catheter stability and local activation time stability. PWI was confirmed by the elimination of local posterior wall electrograms together with activation mapping demonstrating electrical silence within the isolated posterior wall.

### 2.3 Incomplete Lesion Formation

Incomplete lesions were defined as preserved electrical activity and/or conduction gaps confirmed by bipolar electrogram recordings and/or activation mapping. When residual potentials were suspected from 3D voltage map color coding, electrograms from the multipolar mapping catheter were systematically re‐assessed to determine whether residual potentials were truly present. If differentiation from left atrial appendage or right atrial potentials was necessary, pacing from the coronary sinus (for left PVs) or from the superior vena cava (for right PVs) was performed to confirm residual PV potentials. Voltage map color coding was used solely for visualization purposes and not for lesion definition. ICE findings were not used to guide lesion placement or to define incomplete lesions, which were determined solely by electrogram recordings and/or activation mapping. For anatomical analysis, the left atrial PW was divided into subregions as follows: roof region near the LSPV and RSPV, middle‐to‐superior PW, bottom region near the LIPV and RIPV, and middle‐to‐inferior PW. When an incomplete lesion was identified, additional ablation was performed at the same target site in an iterative manner: two applications were delivered, followed by remapping to reassess for residual potentials. If residual conduction persisted, another set of two applications was delivered, with remapping repeated as necessary. Left atrial volume (LAV) was measured using post‐mapping. To identify factors contributing to residual conduction, posterior wall morphology was quantified using the posterior wall bulge distance measured on axial contrast‐enhanced computed tomography (Figure 1). The bulge distance was defined as the perpendicular distance from the line connecting the ostia of the RIPV and LIPV to the most posterior aspect of the left atrial wall. Distances were defined as positive when the posterior wall protruded posteriorly beyond the RIPV–LIPV reference line (convex configuration) and negative when the posterior wall was displaced anteriorly toward the left atrium (concave configuration), typically reflecting compression from adjacent mediastinal structures such as the vertebral body or bronchial tree. Measurements were performed using multiplanar reconstruction by two independent investigators blinded to procedural outcomes.

**Figure 1:**
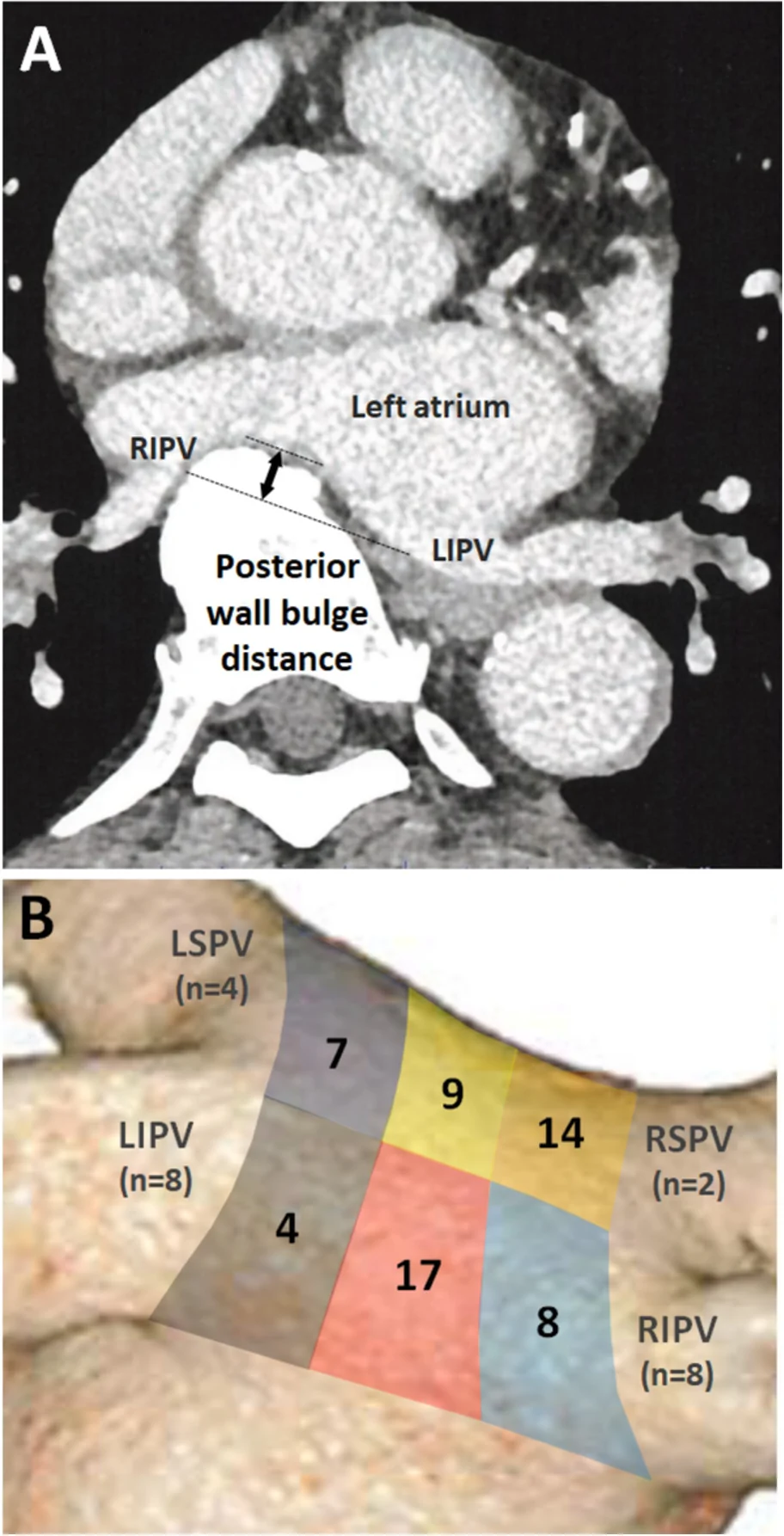
Anatomical and procedural considerations of posterior wall bulging during pulsed‐field ablation. (A) Representative contrast‐enhanced computed tomography image demonstrating posterior wall bulging, defined as the maximal distance from a reference line connecting the inferior pulmonary veins to the posterior wall. (B) Distribution of incomplete lesion locations identified by electroanatomical remapping. Pulmonary vein (PV)‐related incomplete lesions are shown adjacent to each PV. Posterior wall (PW) lesions are divided into six anatomical segments: roof region near the left superior PV (LSPV), roof region near the right superior PV (RSPV), middle‐to‐superior PW, middle‐to‐inferior PW, and bottom regions near the left and right inferior PVs (LIPV and RIPV). The number in each segment represents the frequency of incomplete lesions. The highest frequency was observed in the middle‐to‐inferior PW (*n* = 17), followed by the roof region near the RSPV (*n* = 14).

### 2.4 Learning Curve Analysis

Procedural learning curves were evaluated to characterize temporal changes in the incidence of incomplete lesions requiring additional PFA applications. Procedural sequence was defined chronologically according to case order. To visualize overall temporal trends in procedural performance, locally weighted scatterplot smoothing (LOESS) curves were generated to assess the relationship between case number and the probability of additional PFA applications. To identify the breakpoint of the learning curve and potential transition points in procedural proficiency, we performed a segmented logistic regression analysis (also known as piece‐wise regression or joinpoint analysis). This model was used to estimate the break‐point where the incidence of additional PFA applications significantly changed during the study period. In addition, a 20‐case block incidence analysis was performed to assess the frequency of incomplete lesions within sequential procedural blocks. A 20‐case interval was selected to reduce random case‐to‐case variability and facilitate visualization of temporal trends in lesion completeness while maintaining adequate sample size within each block. Smaller intervals (e.g., 5 or 10 cases) resulted in substantial fluctuation and reduced interpretability of the overall learning trajectory. The incidence of additional PFA applications was calculated as the proportion of cases requiring additional applications within each 20‐case interval. Based on the combined findings from segmented regression and block incidence analysis, procedural maturation was categorized into three phases. The early phase was defined according to the breakpoint identified by segmented regression. The late phase was defined as a 20‐case block incidence of incomplete lesions below 5%. Cases between these two phases were categorized as the intermediate learning phase.

### 2.5 Post‐Procedure Management

The patient underwent continuous electrocardiogram monitoring for 2 days after the procedure. The first outpatient clinic visit was 3 weeks after the procedure. Early AF/atrial tachycardia recurrence was defined as an episode lasting ≥ 30 s occurring within 3 months post‐ablation (blanking period). Follow‐up visits comprised a clinical interview, electrocardiogram, and 24‐h Holter monitoring every 3 months. The last follow‐up entry dataset, which was based on the last office visit, was used to assess the mid‐term freedom from recurrence. Patients were maintained on oral anticoagulants for at least 3 months post‐procedure and thereafter according to the guideline recommendations [3].

### 2.6 Statistical Analysis

Continuous variables are expressed as medians with interquartile ranges (IQR, 25th–75th percentile). Continuous variables were compared using the unpaired *t*‐test or Mann–Whitney U test, as appropriate. The chi‐square or Fisher's exact probability test was used to compare categorical variables. To characterize the procedural learning trajectory, the incidence of acute incomplete lesions was evaluated across sequential cases. A LOESS curve was constructed to visualize temporal trends in incomplete lesion occurrence. In addition, segmented logistic regression analysis was performed to identify potential breakpoints in the relationship between case sequence and the probability of incomplete lesion formation. To further validate the learning curve pattern, the incidence of incomplete lesions was also assessed in sequential 20‐case blocks.

Procedural phases were defined based on the learning curve analysis as early phase, intermediate phase, and late phase. Logistic regression models were used to evaluate determinants of incomplete lesion formation. Variables included in the multivariable model were procedural phase, LAV, and posterior wall bulge distance. Procedural phase was incorporated into the model to account for operator experience and to assess whether anatomical variables independently influenced lesion formation after adjustment for procedural maturation. Arrhythmia recurrence after the blanking period was analyzed using Kaplan–Meier survival analysis, and differences between procedural phases were assessed using the log‐rank test. A two‐tailed *p*‐value < 0.05 was considered statistically significant. Statistical analyses were performed using SPSS Statistics version 30.0 (IBM Corp., Armonk, NY, USA) and R software version 4.5.2 (R Foundation for Statistical Computing, Vienna, Austria) with the “segmented” package.

## 3 Results

### 3.1 Study Population and Overall Incidence of Incomplete Lesions

A total of 182 patients with persistent AF were included in the analysis. Incomplete lesions requiring additional PFA applications were observed in 56 patients (31%), whereas 126 patients (69%) achieved complete lesions without additional applications (Figure 2). Baseline characteristics according to the presence of incomplete lesions are summarized in Table 1. Compared with patients without incomplete lesions, those with incomplete lesions were significantly older (*P* = 0.011), had a higher body mass index (*P* = 0.040), and had a longer duration of continuous AF (*P* < 0.001). Heart failure was also more frequently observed in patients with incomplete lesions (*P* = 0.002). In addition, patients with incomplete lesions had a larger left atrial diameter (*P* = 0.002) and greater LAV on 3D mapping (*P* = 0.032). In contrast, sex distribution, hypertension, diabetes mellitus, prior thromboembolic events, CHADS_2_ score, CHA_2_DS_2_‐VASc score, posterior wall bulge distance, left ventricular ejection fraction, renal function, and serum BNP levels did not differ significantly between the two groups. Procedural parameters according to the presence of incomplete lesions are presented in Table 2. Procedure time and fluoroscopic time differed significantly between groups (both *P* < 0.001). The number of mapping points and time were comparable between groups, whereas the total number of applications was significantly higher in patients with incomplete lesions than that in those without (*P* = 0.011). Procedural complications were rare overall, occurring in 2 patients (1.1%). These included one case of cardiac tamponade and one case of cerebral infarction. Notably, all complications occurred in patients without incomplete lesion formation, whereas no complications were observed in patients with incomplete lesions (0% vs. 1.6%).

**Figure 2:**
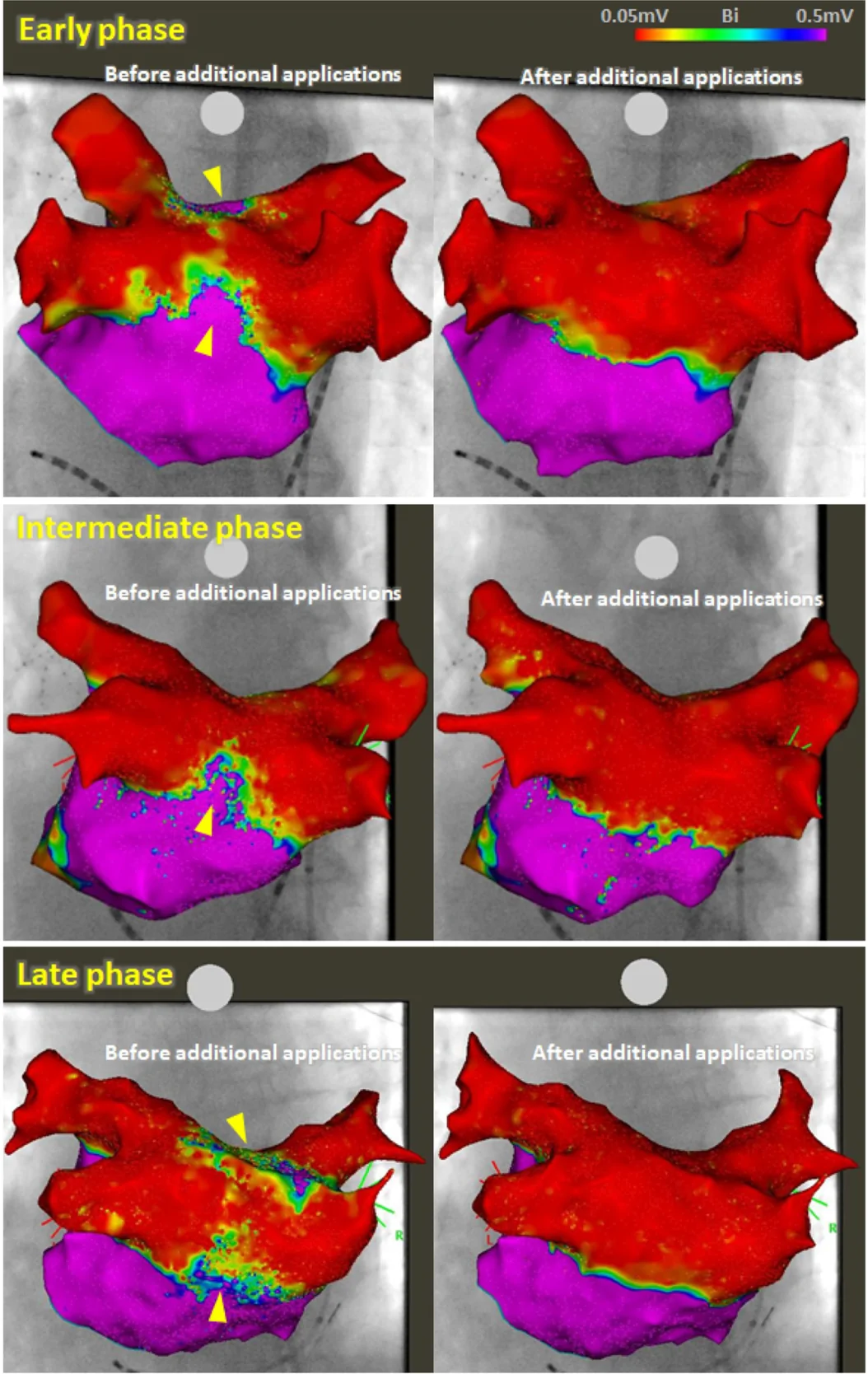
Representative examples of incomplete lesion formation and remapping‐guided correction across procedural phases. Representative voltage maps illustrating incomplete lesion formation and its correction across different procedural phases are shown. Left panels display post‐ablation maps before additional applications, and right panels show maps after additional pulsed‐field applications. In the early phase (upper panels), incomplete lesions (arrowheads) were observed in the middle‐to‐superior and middle‐to‐inferior posterior wall in a patient with a left atrial volume (LAV) of 134.5 cm^3^. In the intermediate phase (middle panels), an incomplete lesion (arrowhead) was observed in the middle‐to‐inferior posterior wall in a patient with an LAV of 110.3 cm^3^. In the late phase (lower panels), small residual incomplete lesions (arrowheads) were observed in the roof near the right superior pulmonary vein and the middle‐to‐inferior posterior wall in a patient with an LAV of 252.5 cm^3^, which may reflect the modest influence of atrial size on lesion formation. These incomplete lesions were effectively eliminated following additional targeted applications, resulting in contiguous low‐voltage lesion sets.

**Table 1:** Baseline characteristics according to the presence of incomplete lesions.

	All (*n* = 182)	Patients without incomplete lesions (*n* = 126)	Patients with incomplete lesions (*n* = 56)	P value
Age (IQR), years	70 (62–77)	73 (63–77)	66 (60–74)	*0.011*
Male, *n* (%)	132 (73)	88 (70)	44 (79)	*0.149*
BMI (IQR), kg/m^2^	23.8 (20.8–26.7)	23.5 (20.5–25.8)	24.7 (22.5–28.0)	*0.040*
Duration of continuous AF (IQR), years	0.3 (0.2–0.7)	0.3 (0.0–0.5)	0.6 (0.3–2.0)	*< 0.001*
Hypertension, *n* (%)	92 (51)	65 (52)	27 (48)	*0.749*
Diabetes Mellitus, *n* (%)	39 (21)	26 (21)	13 (23)	*0.699*
Heart failure, *n* (%)	38 (21)	18 (14)	20 (36)	*0.002*
Prior thromboembolic events, *n* (%)	14 (8)	8 (6)	6 (11)	*0.368*
CHADS_2_ (IQR)	1 (1–2)	1 (1–2)	1 (1–2)	*0.528*
CHA_2_DS_2_‐VASc (IQR)	2 (1–3)	2 (1–3)	2 (1–3)	*0.641*
LA diameter (IQR), mm	41 (38–45)	40 (37–44)	42 (39–49)	*0.002*
LA volume (IQR), cm^3^	188.7 (156.4–215.2)	184.3 (155.3–208.2)	198.4 (159.3–229.9)	*0.032*
LA posterior wall bulge distance	−2.1 (−4.0‐1.8)	−2.0 (−4.2‐2.0)	−2.4 (−4.0‐1.5)	*0.849*
Left ventricular ejection fraction (IQR), %	61 (53–67)	60 (55–67)	61 (44–68)	*0.827*
Estimated GFR (IQR), mL/min/1.73 m^2^	57.0 (48.0–66.2)	56.0 (48.0–65.0)	58.5 (48.0–70.0)	*0.300*
Serum BNP (IQR), pg/mL	147.7 (93.1–238.3)	142.2 (87.9–232.9)	178.3 (109.7–245.5)	*0.182*

Abbreviations: AF, atrial fibrillation; BMI, body mass index; BNP, brain natriuretic peptide; IQR, interquartile range; LA, left atrial.

**Table 2:** Procedural parameters and complications according to the presence of incomplete lesions.

	All (*n* = 182)	Patients without incomplete lesions (*n* = 126)	Patients with incomplete lesions (*n* = 56)	P value
Procedure time (IQR), min	60 (50–75)	60.0 (50.0–70.0)	70.0 (60.0–87.0)	*< 0.001*
Fluoroscopic time (IQR), min	10.0 (7.2–12.3)	8.0 (7.0–11.0)	12.5 (10.0‐–18.0)	*< 0.001*
Mapping points (IQR)	5182 (3625–6688)	5475 (3643–6735)	4759 (3476–6299)	*0.237*
Mapping time (IQR), min	7.0 (6.0–9.0)	7.0 (6.0–9.0)	8.0 (6.0–9.3)	*0.330*
No. of applications	54 (50–76)	54 (50–58)	56 (51–64)	*0.011*
Procedural complications, *n* (%)	2 (1.1)	2 (1.6)	0 (0)	*NS*

Abbreviations: IQR, interquartile range.

Incomplete lesion formation identified by electroanatomical remapping was categorized into PV–related lesions and PW lesions (Figure 1). PV‐related incomplete lesions were observed in the LIPV (*n* = 8), RIPV (*n* = 8), LSPV (*n* = 4), and RSPV (*n* = 2). PW‐related incomplete lesions were most frequently observed in the middle‐to‐inferior PW (*n* = 17), followed by the roof region near the RSPV (*n* = 14). Other locations included the middle‐to‐superior PW (*n* = 9), the bottom region near the RIPV (*n* = 8), the roof region near the LSPV (*n* = 7), and the bottom region near the LIPV (*n* = 4). Although acute incomplete lesions requiring additional PFA applications were identified during systematic electroanatomical remapping, complete acute PWI was ultimately achieved in all patients following additional PFA applications.

Repeat ablation was performed in 11 patients because of recurrent atrial arrhythmias. Pulmonary vein reconnection was identified in seven patients and posterior wall reconnection in six. Three patients required additional PFA applications because of acute incomplete lesions during the index procedure. In two of these patients, reconnection occurred at or adjacent to the previously identified acute gap sites, whereas one patient demonstrated reconnection at a different location (Supporting Information S1: Table 1). Because of the limited number of repeat procedures, no definitive relationship between acute incomplete lesions and chronic lesion durability could be established.

### 3.2 Learning Curve Analysis

LOESS analysis demonstrated a progressive decline in incomplete lesion formation with increasing procedural experience. Segmented logistic regression identified an estimated breakpoint at 19.6 cases (95% CI: 0–43.1), suggesting that the greatest reduction in incomplete lesion formation occurred during the initial phase of procedural adoption (Figure 3). Thereafter, incomplete lesions became less frequent but were not completely eliminated. A supplementary 20‐case block analysis showed that the incidence of incomplete lesions fell below 5% beyond approximately 160 cases (Supporting Information S1: Figure 2).

**Figure 3:**
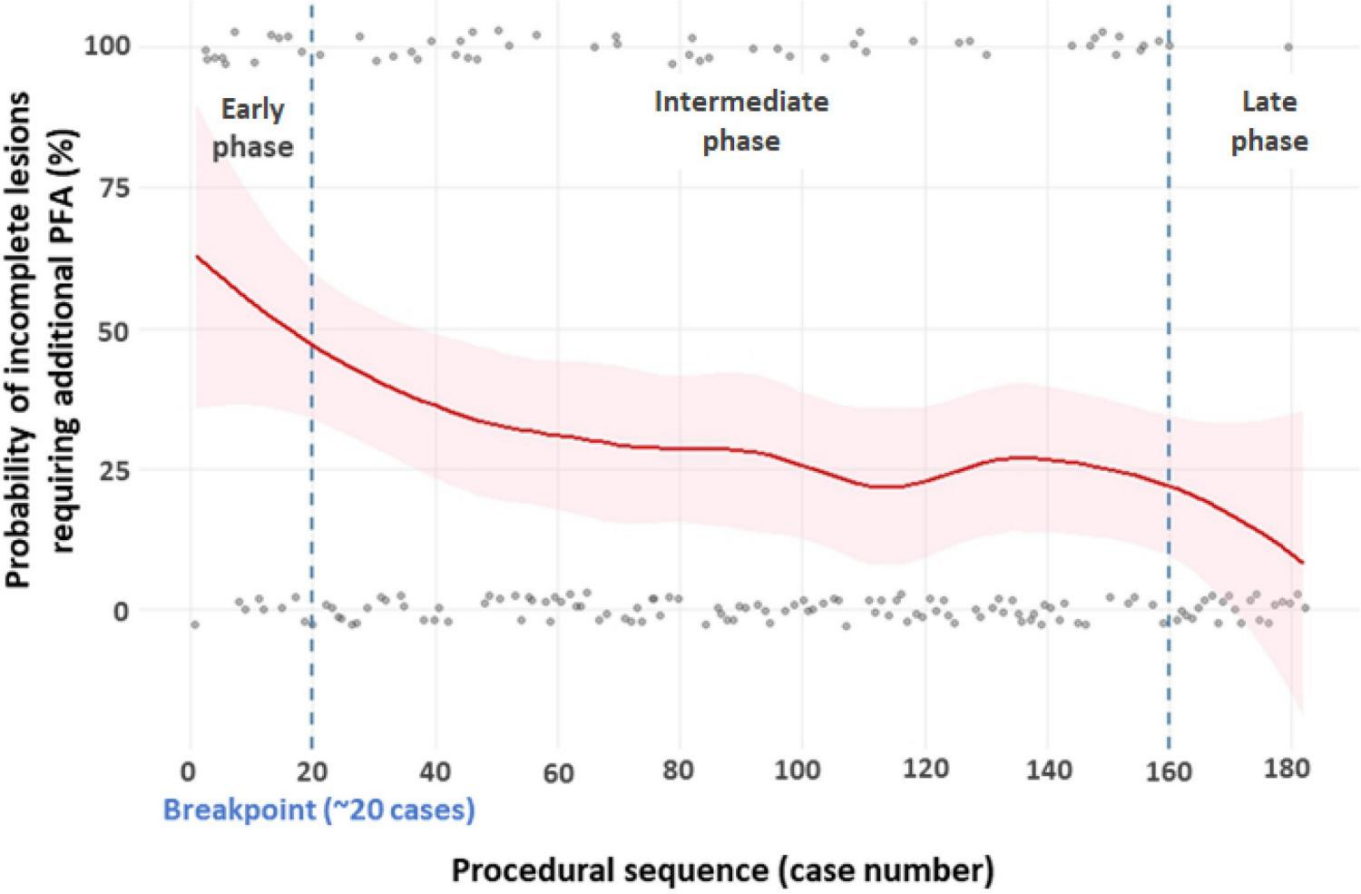
Procedural learning curve of pentaspline pulsed‐field ablation with posterior wall isolation. Scatter plot with a locally weighted scatterplot smoothing (LOESS) curve demonstrating the probability of incomplete lesions requiring additional pulsed‐field applications across the procedural sequence. The shaded area represents the 95% confidence interval. A steep decline in incomplete lesion formation was observed during the initial phase of procedural adoption, followed by more gradual improvement with increasing procedural experience.

For exploratory analyses, procedures were categorized into an early phase (cases 1–20), an intermediate phase (cases 21–160), and a late phase (≥ 161 cases). Baseline characteristics across procedural phases are shown in Table 3. No significant differences in demographic or clinical characteristics were observed among the three groups. Procedural characteristics according to procedural phase are presented in Table 4. Procedure time and fluoroscopic time differed significantly across phases (both *p* < 0.001). The number of mapping points increased significantly with procedural experience (*p* < 0.001), whereas mapping time remained comparable across phases. The total number of PFA applications also increased significantly with procedural experience (*p* < 0.001), suggesting progressive refinement of lesion deployment strategies. Compared with the late phase, the risk of incomplete lesions was significantly higher during earlier procedural phases. In multivariable logistic regression analysis, the early phase was associated with a 24.4‐fold higher risk of incomplete lesion formation (95% CI 2.73–219.10; *p* = 0.004), whereas the intermediate phase demonstrated a 9.26‐fold higher risk (95% CI 1.20–71.24; *p* = 0.032).

**Table 3:** Baseline characteristics across the three procedural learning phases.

	All (*n* = 182)	Early phase (1–20)	Intermediate phase (21–160)	Late phase (161–182)	P value
Age (IQR), years	70 (62–77)	71 (63–76)	68 (61–77)	73 (63–79)	*0.551*
Male, *n* (%)	132 (73)	13 (65)	105 (75)	14 (64)	*0.392*
BMI (IQR), kg/m^2^	23.8 (20.8–26.7)	24.6 (20.8–26.9)	23.7 (21.0–26.9)	23.2 (20.0–25.6)	*0.694*
Duration of continuous AF (IQR), years	0.3 (0.2–0.7)	0.4 (0.3–1.3)	0.3 (0.1–0.7)	0.3 (0.2–0.7)	*0.272*
Hypertension, *n* (%)	92 (51)	12 (60)	68 (49)	12 (55)	*0.584*
Diabetes Mellitus, *n* (%)	39 (21)	8 (40)	28 (20)	3 (14)	*0.080*
Heart failure, *n* (%)	38 (21)	5 (25)	30 (21)	3 (14)	*0.628*
Prior thromboembolic events, *n* (%)	14 (8)	1 (5)	12 (9)	1 (5)	*0.718*
CHADS_2_ (IQR)	1 (1‐2)	2 (1‐2)	1 (1‐2)	2 (1‐2)	*0.319*
CHA_2_DS_2_‐VASc (IQR)	2 (1–3)	3 (2–4)	2 (1–3)	3 (2–3)	*0.254*
LAD (IQR), mm	41 (38–45)	41 (37–50)	41 (38–45)	41 (38–45)	*0.758*
LA volume (IQR), cm^3^	188.7 (156.4–215.2)	183.8 (141.4–226.6)	187.6 (156.2–214.6)	192.3 (176.4–215.7)	*0.778*
LA posterior wall bulge distance	−2.1 (−4.0‐1.8)	−2.9 (−5.4‐1.2)	−2.0 (−4.0‐2.1)	−3.4 (−4.6‐1.2)	*0.134*
Left ventricular EF (IQR), %	61 (53–67)	65 (58–67)	61 (52–68)	59 (54–62)	*0.203*
Estimated GFR (IQR), mL/min/1.73 m^2^	57.0 (48.0–66.2)	56.0 (40.5–62.5)	56.0 (48.0–67.8)	58.5 (50.2–63.0)	*0.813*
Serum BNP (IQR), pg/mL	147.7 (93.1–238.3)	159.4 (103.2–333.9)	143.4 (90.0–236.3)	165.6 (91.3–244.7)	*0.747*

Abbreviations: AF, atrial fibrillation; BMI, body mass index; BNP, brain natriuretic peptide; EF, ejection fraction; IQR, interquartile range; LAD, left atrial diameter.

**Table 4:** Procedural parameters according to learning phase.

	All (*n* = 182)	Early phase (1–20)	Intermediate phase (21–160)	Late phase (161–182)	P value
Procedure time (IQR), min	60 (50–75)	81 (69–98)	60 (50–70)	69 (61–81)	*< 0.001*
Fluoroscopic time (IQR), min	10.0 (7.2–12.3)	12.0 (10.3–20.0)	9.0 (7.0–12.0)	9.0 (7.0–13.3)	*< 0.001*
Mapping points (IQR)	5182 (3625–6688)	4629 (3503–6253)	4796 (3344–6483)	6997 (5807–7756)	*< 0.001*
Mapping time (IQR), min	7.0 (6.0–9.0)	7.0 (5.0–9.8)	7.0 (6.0–9.0)	8.0 (6.0–10.0)	*0.329*
No. of applications	54 (50–6)	48 (46–51)	54 (51–60)	58 (56–62)	*< 0.001*

Abbreviation: IQR, interquartile range.

A sensitivity analysis focusing specifically on PW incomplete lesions yielded similar findings. In multivariable logistic regression analysis, procedural phase remained strongly associated with PW incomplete lesions. Compared with the late phase, the early phase was associated with a significantly higher risk of PW incomplete lesions (OR 18.23, 95% CI 1.91–174.17; *p* = 0.012), whereas the intermediate phase showed a trend toward increased risk (OR 7.13, 95% CI 0.91–55.78; *p* = 0.061).

### 3.3 Anatomical Determinants of Incomplete Lesions

Multivariable analysis revealed that procedural phase remained the dominant determinant of incomplete lesions. LAV was independently but modestly associated with incomplete lesions (OR 1.009 per mL; *p* = 0.021). In contrast, posterior wall bulge distance was not independently associated with incomplete lesion formation. A sensitivity analysis focusing on posterior wall incomplete lesions showed a borderline association with LAV (OR 1.008 per mL, 95% CI 1.000–1.017; *p* = 0.057), whereas posterior wall bulge distance was not associated (OR 1.016, 95% CI 0.922–1.120; *p* = 0.746).

### 3.4 Arrhythmia Outcomes Across Procedural Phases

The median follow‐up duration was 6.2 months (interquartile range: 3.8–9.1 months), with shorter follow‐up in the late phase due to the chronological nature of the study cohort. Arrhythmia recurrence did not differ across phases; however, follow‐up duration was shorter in the late phase. (Log‐rank *P* = 0.488). An additional analysis using an 8‐week blanking period yielded identical results to those obtained using the conventional 3‐month blanking period, as no patient experienced a first documented atrial arrhythmia recurrence between 8 weeks and 3 months after the index procedure.

## 4 Discussion

The principal findings of the present study are as follows. First, pentaspline PFA with PWI demonstrated a learning trajectory characterized by a marked reduction in incomplete lesion formation during the initial phase of procedural adoption, with the greatest improvement occurring within the first approximately 20 procedures. Second, procedural experience emerged as the dominant determinant of lesion completeness, whereas anatomical factors exerted a comparatively limited influence; left atrial volume showed a modest independent association with incomplete lesion formation, whereas posterior wall geometry was not independently associated. Third, residual conduction gaps were not completely eliminated despite increasing procedural experience, highlighting the continued importance of systematic electroanatomical remapping for identifying and correcting incomplete lesions. Finally, mid‐term arrhythmia recurrence did not differ across procedural phases, although interpretation of this finding is limited by the shorter follow‐up duration in the later phase of the study.

### 4.1 Learning Curve of Pentaspline PFA

Learning curves have been well described for conventional AF ablation technologies. Prior studies demonstrated progressive improvements in procedural efficiency and clinical outcomes with increasing operator experience during radiofrequency ablation, emphasizing the importance of cumulative procedural exposure [13]. Similar trends have been reported for cryoballoon ablation, where procedural success improves as operators gain familiarity with catheter manipulation and lesion deployment strategies [14]. More recently, early experience with PFA has suggested relatively rapid procedural adoption compared with conventional thermal ablation modalities, with high acute PVI rates and favorable safety profiles reported even during early clinical use [15, 16]. However, these studies primarily focused on PVI and procedural workflow metrics rather than detailed evaluation of lesion completeness. In addition, recent analyses have suggested that operator experience continues to influence procedural performance beyond the initial adoption phase [17]. In contrast, the present study specifically quantified the learning trajectory for combined PVI and PWI using systematic high‐density electroanatomical remapping. The greatest improvement in lesion completeness occurred during the early phase of procedural adoption, corresponding to approximately the first 20 procedures. Thereafter, lesion completeness continued to improve with increasing experience, although residual conduction gaps were not completely eliminated. These findings indicate that procedural experience progressively improves lesion quality but does not entirely obviate the occurrence of incomplete lesions. Accordingly, systematic remapping remains important even after substantial procedural experience has been accumulated. Furthermore, the persistence of incomplete lesions despite increasing experience suggests that factors beyond static anatomical characteristics and operator familiarity may continue to influence lesion formation. Although PWI was confirmed using activation mapping and elimination of local electrograms, exit block pacing was not performed systematically. Because bidirectional block assessment would provide stronger evidence of complete electrical isolation, future prospective studies incorporating both entrance and exit block assessment are warranted.

### 4.2 Anatomical Determinants: Experience Over Geometry

In addition to procedural experience, anatomical factors may influence lesion formation during AF ablation. In the present study, LAV demonstrated a modest independent association with incomplete lesion formation, suggesting a limited anatomical influence that persists despite increasing operator experience. Larger atrial chambers may require broader lesion coverage and may increase the likelihood of residual conduction, particularly during PWI. In contrast, posterior wall morphology assessed by bulge distance was not independently associated with incomplete lesions. Importantly, this finding remained consistent even when the analysis was restricted to posterior wall lesions, excluding cases with pulmonary vein–only incomplete lesions.

These results indicate that static anatomical geometry alone does not adequately explain lesion continuity in pentaspline PFA. Rather, lesion completeness may depend more heavily on dynamic procedural factors, including catheter positioning, tissue contact, and lesion overlap. Taken together, the findings suggest that procedural experience exerts a greater influence on lesion completeness than the anatomical parameters evaluated in this study. Mechanistically, increasing operator experience may improve catheter orientation, tissue contact optimization, and lesion overlap strategies, thereby reducing the likelihood of conduction gaps. Consistent with this interpretation, the number of PFA applications increased with procedural experience, which may reflect progressive refinement of lesion deployment strategies to accommodate anatomical variability.

### 4.3 Clinical Outcomes and the Role of Remapping

From a practical perspective, the present findings suggest that the greatest improvement in lesion completeness occurs during the initial phase of procedural adoption, with a marked reduction in incomplete lesion formation during the first approximately 20 procedures. Thereafter, lesion completeness continued to improve with increasing experience, although residual conduction gaps were not completely eliminated. These observations indicate that procedural experience progressively improves lesion quality but does not entirely obviate the occurrence of incomplete lesions. Accordingly, systematic remapping should be considered an integral component of lesion assessment rather than a temporary adjunct used only during the learning phase.

The persistence of residual conduction gaps despite substantial procedural experience suggests that some lesions may remain difficult to assess using conventional fluoroscopy‐ and ICE‐guided workflows alone. Technologies enabling real‐time assessment of catheter configuration and tissue contact, such as the latest generation of FARAVIEW, may help address this limitation and further improve lesion completeness. Future studies are warranted to determine whether these advances can reduce incomplete lesion formation beyond the improvements achieved through procedural experience alone.

To further explore the clinical relevance of acute incomplete lesions, we reviewed repeat ablation findings. Although reconnection occurred at or adjacent to previously identified acute gap sites in some patients, these observations suggest that regions demonstrating acute incomplete lesions may represent anatomically or procedurally challenging sites. Conversely, because all acute incomplete lesions were identified and corrected during the index procedure, systematic remapping with additional targeted applications may also have prevented chronic reconnection at other acute gap sites that would otherwise have persisted. Taken together, these findings suggest that acute incomplete lesions should not simply be regarded as failed lesions, but rather as indicators of regions requiring careful assessment and additional lesion deployment during the index procedure. Given the limited number of repeat procedures, these observations should be considered hypothesis‐generating and require confirmation in larger prospective studies. Therefore, the principal implication of the present study is that systematic electroanatomical remapping improves acute lesion quality by identifying residual conduction gaps and enabling immediate corrective ablation rather than merely improving procedural efficiency. Whether optimization of acute lesion quality ultimately translates into improved lesion durability and better long‐term clinical outcomes warrants further investigation.

The present study should not be interpreted as supporting routine empirical PWI in all patients with persistent AF. Rather, our findings demonstrate that when PWI is selected as part of the ablation strategy, systematic electroanatomical remapping facilitates identification and correction of residual conduction gaps, thereby improving acute lesion quality. Whether empirical PWI itself provides incremental clinical benefit remains controversial. Recent subgroup analyses from the MANIFEST‐PF registry [10] and the EU‐PORIA registry [18] have questioned the additional benefit of empirical PWI using single‐shot PFA systems. Accordingly, the principal implication of our findings is optimization of acute lesion assessment and acute lesion quality rather than justification of routine empirical PWI. Given the potential trade‐off between more extensive lesion sets and procedure‐related risks, the decision to perform empirical PWI should be individualized until its incremental clinical benefit is established.

### 4.4 Limitations

Several limitations should be acknowledged. First, this was a single‐center observational study conducted under a standardized procedural workflow with direct supervision by a senior operator. Accordingly, the observed learning trajectory should be interpreted as reflecting procedural maturation at the program level rather than an individual operator learning curve. The findings therefore may not be directly generalizable to institutions with different operator experience, supervision models, or procedural workflows. Second, the study focused on acute lesion completeness assessed by intra‐procedural high‐density remapping, and long‐term lesion durability was not directly evaluated. Although remapping enabled real‐time identification and correction of residual conduction, the extent to which these acute findings translate into durable lesion sets and long‐term clinical outcomes requires further investigation. In addition, follow‐up duration was shorter in the later phase of the study, limiting interpretation of phase‐specific recurrence outcomes. Third, although anatomical parameters were assessed using preprocedural computed tomography, additional structural or functional factors—such as tissue thickness, catheter–tissue contact dynamics, and electrophysiological properties—were not directly evaluated and may also influence lesion formation. In addition, the range of posterior wall morphologies in the present cohort was relatively limited, with predominantly flat or mildly concave posterior wall configurations. Therefore, the ability to detect an independent effect of posterior wall geometry may have been limited. Finally, the present findings reflect experience with a specific pentaspline PFA platform and a fluoroscopy‐ and ICE‐guided workflow. Therefore, the observed learning trajectory and determinants of incomplete lesion formation may not be directly applicable to other catheter designs, energy delivery systems, or emerging visualization technologies.

## 5 Conclusion

Pentaspline PFA with posterior wall isolation for persistent atrial fibrillation demonstrated progressive improvement in acute lesion completeness with increasing procedural experience. The most substantial reduction in incomplete lesion formation occurred during the early phase of procedural adoption, whereas anatomical factors contributed only modestly. Although lesion completeness improved over time, residual conduction gaps were not completely eliminated, underscoring the continued importance of systematic electroanatomical remapping for identifying and correcting incomplete lesions. These findings provide practical insights into procedural maturation during adoption of pentaspline pulsed‐field ablation and support the integration of systematic electroanatomical remapping for lesion assessment throughout the learning process. Further multicenter studies with longer follow‐up are warranted to confirm these observations and determine whether emerging visualization technologies can further improve lesion completeness beyond the effects of procedural experience alone.

## Funding

The authors have nothing to report.

## Ethics Statement

This study was conducted in accordance with the principles of the Declaration of Helsinki regarding investigations in humans and was approved by the Institutional Ethics Committee of Tokai University Hospital (approval no. 25R209).

## Conflicts of Interest

The authors declare no conflicts of interest.

## Data Availability Statement

The data that support the findings of this study are available on request from the corresponding author. The data are not publicly available due to privacy or ethical restrictions.

## Supporting information


**Figure S1:** Continuous sequential deployment strategy (“single‐stroke” workflow) for posterior wall isolation. (A) Sequential fluoroscopic images illustrating the continuous sequential deployment of the pentaspline catheter during posterior wall isolation. The catheter is advanced in a continuous inverted U‐shaped trajectory with overlapping applications to create a contiguous posterior wall lesion set. Arrows indicate the direction of catheter movement. **(B)** Corresponding electroanatomical map demonstrating the contiguous posterior wall lesion set created using this continuous sequential deployment strategy

**Figure S2:** Sequential 20‐case block incidence analysis of incomplete lesion formation. Incidence of incomplete lesions requiring additional pulsed‐field applications according to procedural sequence in consecutive 20‐case blocks. Although variability was observed between individual blocks, the overall trend demonstrated progressive reduction in incomplete lesion formation with increasing procedural experience. The incidence fell below 5% after approximately 160 cases; however, this threshold should be interpreted as an exploratory indicator of procedural stabilization rather than a definitive benchmark

Supporting File
