# Supplementary material for: Learning Curve of Pentaspline Pulsed‐Field Ablation With Posterior Wall Isolation for Persistent Atrial Fibrillation
Source: J Cardiovasc Electrophysiol. 2026 Sep 27;37(11):2698–708. doi: 10.1111/jce.70515 (PMC13768788; doi:10.1111/jce.70515)
Supplement: Supplementary file 1 [file JCE-37-2698-s003.docx]

**Supplementary Table 1. Repeat ablation findings in patients undergoing repeat procedures**

| Case | Acute incomplete lesion (index procedure) | Acute gap location | Reconnected PV(s) | Reconnected PW region(s) | Correspondence with acute gap |
| --- | --- | --- | --- | --- | --- |
| 1 | No | — | RSPV | Middle-to-superior PW, Middle-to-inferior PW | N/A |
| 2 | No | — | RSPV | Middle-to-superior PW | N/A |
| 3 | No | — | RIPV, LSPV, LIPV | None | N/A |
| 4 | No | — | RSPV | None | N/A |
| 5 | Yes | RIPV | LSPV, LIPV | Middle-to-superior PW, Middle-to-inferior PW, Bottom near LIPV | No |
| 6 | No | — | None | None | N/A |
| 7 | Yes | Middle-to-superior PW, Middle-to-inferior PW | RSPV | Middle-to-superior PW, Middle-to-inferior PW | Yes |
| 8 | No | — | None | Middle-to-superior PW, Middle-to-inferior PW | N/A |
| 9 | No | — | RSPV, LSPV | None | N/A |
| 10 | No | — | None | Roof near LSPV, Middle-to-superior PW | N/A |
| 11 | Yes | Bottom near RIPV | None | Roof near RSPV, Bottom near RIPV | Yes |

Complete acute posterior wall isolation was achieved in all patients during the index procedure following additional PFA applications when required. "Correspondence with acute gap" indicates that chronic reconnection was identified at the same or an anatomically adjacent site as the acute incomplete lesion observed during the index procedure.

Abbreviations: LIPV, left inferior pulmonary vein; LSPV, left superior pulmonary vein; PFA, pulsed-field ablation; PV, pulmonary vein; PW, posterior wall; RIPV, right inferior pulmonary vein; RSPV, right superior pulmonary vein.
